# *N*-(Imidazol-1-ylmeth­yl)phthalimide

**DOI:** 10.1107/S1600536808025154

**Published:** 2008-08-13

**Authors:** Su-Qing Wang, Fang-Fang Jian, Huan-Qiang Liu

**Affiliations:** a Microscale Science Institute, Department of Chemistry and Chemical Engineering, Weifang University, Weifang 261061, People’s Republic of China; b Department of Chemistry and Chemical Engineering, Weifang University, Weifang 261061, People’s Republic of China

## Abstract

The title compound [systematic name: 2-(imidazol-1-ylmeth­yl)isoindole-1,3-dione], C_12_H_9_N_3_O_2_, was prepared by reaction of *N*-(bromo­meth­yl)phthalimide and imidazole in chloro­form solution. The crystal structure is stabilized by weak inter­molecular C—H⋯π inter­actions and inter­molecular π–π inter­actions with centroid–centroid distances in the range 3.6469 (8)–3.8831 (9) Å.

## Related literature

For related literature, see: Brooks & Davidson (1960 ▶); Zhao *et al.* (2000 ▶); Barszcz *et al.* (2004 ▶); Jian *et al.* (2004 ▶).
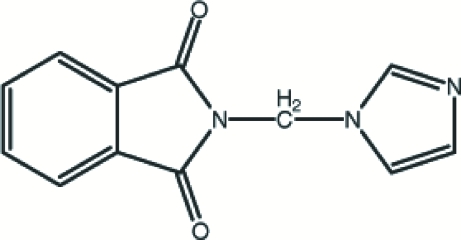


## Experimental

### 

#### Crystal data

C_12_H_9_N_3_O_2_
*M* *_r_* = 227.22
Monoclinic, 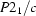

*a* = 7.9905 (6) Å
*b* = 19.8096 (15) Å
*c* = 6.9229 (5) Å
β = 105.5540 (10)°
*V* = 1055.69 (14) Å^3^
*Z* = 4
Mo *K*α radiation
μ = 0.10 mm^−1^
*T* = 273 (2) K
0.2 × 0.15 × 0.15 mm

#### Data collection

Bruker SMART CCD area-detector diffractometer
Absorption correction: none
6792 measured reflections
2556 independent reflections
1868 reflections with *I* > 2σ(*I*)
*R*_int_ = 0.027

#### Refinement

*R*[*F* ^2^ > 2σ(*F* ^2^)] = 0.039
*wR*(*F* ^2^) = 0.101
*S* = 1.03
2556 reflections
155 parameters
H-atom parameters constrained
Δρ_max_ = 0.24 e Å^−3^
Δρ_min_ = −0.15 e Å^−3^

### 

Data collection: *SMART* (Bruker, 1997 ▶); cell refinement: *SAINT* (Bruker, 1997 ▶); data reduction: *SAINT*; program(s) used to solve structure: *SHELXS97* (Sheldrick, 2008 ▶); program(s) used to refine structure: *SHELXL97* (Sheldrick, 2008 ▶); molecular graphics: *SHELXTL* (Sheldrick, 2008 ▶); software used to prepare material for publication: *SHELXTL*.

## Supplementary Material

Crystal structure: contains datablocks global, I. DOI: 10.1107/S1600536808025154/at2605sup1.cif

Structure factors: contains datablocks I. DOI: 10.1107/S1600536808025154/at2605Isup2.hkl

Additional supplementary materials:  crystallographic information; 3D view; checkCIF report

## Figures and Tables

**Table 1:** Selected interatomic distances (Å) *Cg*1, *Cg*2 and *Cg*3 are the centroids of the N1/N3/C1–C3, N3/C5/C6/C11/C12 and C6–C11 rings, respectively.

*Cg*1⋯*Cg*1^i^	3.8831 (9)
*Cg*2⋯*Cg*3^ii^	3.6985 (8)
*Cg*2⋯*Cg*3^iii^	3.6469 (8)
*Cg*3⋯*Cg*3^ii^	3.7214 (8)

Symmetry codes: (i) 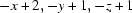; (ii) 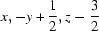; (iii) 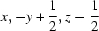.

**Table 2:** Hydrogen-bond geometry (Å, °) *Cg*1 is the centroid of the N1/N3/C1–C3 ring.

*D*—H⋯*A*	*D*—H	H⋯*A*	*D*⋯*A*	*D*—H⋯*A*
C10—H10*A*⋯*Cg*1^iv^	0.93	2.94	3.6105 (15)	130

Symmetry code: (iv) 
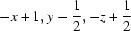
.

**Fig. 1.**
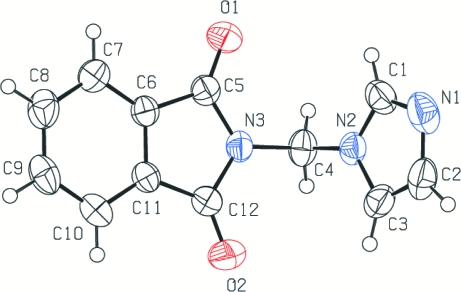
The molecular structure and atom-labeling scheme for (I), with displacement ellipsoids drawn at the 50% probability level.

**Fig. 2.**
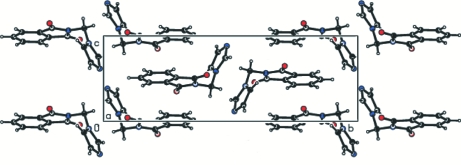
The crystal packing of (I), viewed down the a axis.

## supplementary crystallographic information

### Comment

The imidazole and its derivatives are of considerable interest as the ligands in many biological systems in which they provide the potential binding site for metal ions (Brooks *et al.*, 1960). In our search for new ligands of this type, we have synthesized the title compound (I), and describe its structure here.

In the crystal structure of (I) (Fig. 1), the C═O bond lengths are 1.2030 (15) and 1.2026 (15) Å. The C—N bond lengths are in agreement with those observed before (Zhao *et al.*, 2000). The dihedral angles formed by the N1/N2/C1-C3 and N3/C5/C6/C11/C12 rings, with the C6-C12 ring, are 70.95 (7) ang 0.44 (7)°, respectively.

The crystal structure is stabilized by weak intermolecular C—H···π interactions and intermolecular π–π interactions with centroid-to-centroid distances of 3.6469 (8)–3.8831 (9) Å (Table 1). The crystal packing of the title compound is shown in Fig. 2, viewed down the *a* axis.

### Experimental

*N*-bromomethyl phthalic imidine 7.2 g (0.03 mol) and imidazole 2.04 g (0.03 mol) were dissolved in 20 ml chloroform. The solution was cooled to 283 K. Then, 3 g (0.03 mol) triethylamine was added dropwise *via* cannula into the well stirred solution.The reaction mixture was stirred at 283 K for 6 h. Then the solution was continued to stir at room temperature about 17 h. 20 ml water was added into the solution, the organic phase was seperated and dryed with anhydrous potassium carbonate. The colourless organic phase was evaporated. The title compound is afforded in 65% yield. The colourless crystals of suitable for X-ray determination were obtained from anhydrous ethanol at room temperature after two days.

### Refinement

H atoms were fixed geometrically and allowed to ride on their parent atoms, with C—H distance of 0.93 Å, respectively, and with *U*_iso_=1.2*U*_eq_ of the parent atoms.

### Crystal data

**Table:**

C_12_H_9_N_3_O_2_	*F*_000_ = 472
*M**_r_* = 227.22	*D*_x_ = 1.430 Mg m^−^^3^
Monoclinic, *P*2_1_/*c*	Mo *K*α radiation λ = 0.71073 Å
Hall symbol: -P 2ybc	Cell parameters from 2256 reflections
*a* = 7.9905 (6) Å	θ = 2.1–28.3º
*b* = 19.8096 (15) Å	µ = 0.10 mm^−^^1^
*c* = 6.9229 (5) Å	*T* = 273 (2) K
β = 105.5540 (10)º	Bar, colourless
*V* = 1055.69 (14) Å^3^	0.2 × 0.15 × 0.15 mm
*Z* = 4	

### Data collection

**Table:**

Bruker SMART CCD area-detector diffractometer	1868 reflections with *I* > 2σ(*I*)
Radiation source: fine-focus sealed tube	*R*_int_ = 0.027
Monochromator: graphite	θ_max_ = 28.3º
*T* = 293(2) K	θ_min_ = 2.1º
φ and ω scans	*h* = −7→10
Absorption correction: none	*k* = −25→26
6792 measured reflections	*l* = −8→9
2556 independent reflections	

### Refinement

**Table:**

Refinement on *F*^2^	Hydrogen site location: inferred from neighbouring sites
Least-squares matrix: full	H-atom parameters constrained
*R*[*F*^2^ > 2σ(*F*^2^)] = 0.039	*w* = 1/[σ^2^(*F*_o_^2^) + (0.0428*P*)^2^ + 0.1392*P*] where *P* = (*F*_o_^2^ + 2*F*_c_^2^)/3
*wR*(*F*^2^) = 0.101	(Δ/σ)_max_ < 0.001
*S* = 1.03	Δρ_max_ = 0.24 e Å^−^^3^
2556 reflections	Δρ_min_ = −0.15 e Å^−^^3^
155 parameters	Extinction correction: SHELXL97 (Sheldrick, 2008), Fc^*^=kFc[1+0.001xFc^2^λ^3^/sin(2θ)]^-1/4^
Primary atom site location: structure-invariant direct methods	Extinction coefficient: 0.008 (2)
Secondary atom site location: difference Fourier map	

### Special details

**Table:**

Geometry. All e.s.d.'s (except the e.s.d. in the dihedral angle between two l.s. planes) are estimated using the full covariance matrix. The cell e.s.d.'s are taken into account individually in the estimation of e.s.d.'s in distances, angles and torsion angles; correlations between e.s.d.'s in cell parameters are only used when they are defined by crystal symmetry. An approximate (isotropic) treatment of cell e.s.d.'s is used for estimating e.s.d.'s involving l.s. planes.
Refinement. Refinement of *F*^2^ against ALL reflections. The weighted *R*-factor *wR* and goodness of fit *S* are based on *F*^2^, conventional *R*-factors *R* are based on *F*, with *F* set to zero for negative *F*^2^. The threshold expression of *F*^2^ > σ(*F*^2^) is used only for calculating *R*-factors(gt) *etc*. and is not relevant to the choice of reflections for refinement. *R*-factors based on *F*^2^ are statistically about twice as large as those based on *F*, and *R*- factors based on ALL data will be even larger.

### Fractional atomic coordinates and isotropic or equivalent isotropic displacement parameters (Å^2^)

**Table:**

	*x*	*y*	*z*	*U*_iso_*/*U*_eq_	
O1	0.38144 (13)	0.59386 (5)	0.45690 (16)	0.0582 (3)	
O2	0.88708 (13)	0.71264 (5)	0.64832 (19)	0.0713 (3)	
N1	0.79858 (19)	0.51519 (6)	0.1175 (2)	0.0656 (4)	
N2	0.80767 (14)	0.55831 (5)	0.41391 (16)	0.0424 (3)	
N3	0.65627 (13)	0.63862 (5)	0.56497 (15)	0.0407 (3)	
C1	0.7178 (2)	0.52112 (7)	0.2578 (2)	0.0535 (4)	
H1B	0.6103	0.5018	0.2505	0.064*	
C2	0.9506 (2)	0.55038 (7)	0.1900 (3)	0.0604 (4)	
H2A	1.0367	0.5550	0.1234	0.073*	
C3	0.95804 (18)	0.57721 (7)	0.3700 (2)	0.0518 (4)	
H3A	1.0472	0.6033	0.4488	0.062*	
C4	0.75385 (19)	0.57605 (6)	0.5912 (2)	0.0471 (3)	
H4A	0.8558	0.5806	0.7042	0.057*	
H4B	0.6827	0.5400	0.6216	0.057*	
C5	0.47554 (17)	0.64220 (6)	0.49665 (18)	0.0402 (3)	
C6	0.43317 (15)	0.71539 (6)	0.48572 (17)	0.0382 (3)	
C7	0.27516 (18)	0.74781 (7)	0.4294 (2)	0.0510 (3)	
H7A	0.1717	0.7237	0.3900	0.061*	
C8	0.2764 (2)	0.81774 (8)	0.4338 (2)	0.0582 (4)	
H8A	0.1715	0.8410	0.3980	0.070*	
C9	0.4295 (2)	0.85355 (7)	0.4899 (2)	0.0540 (4)	
H9A	0.4259	0.9005	0.4911	0.065*	
C10	0.58863 (18)	0.82107 (7)	0.54462 (18)	0.0476 (3)	
H10A	0.6922	0.8452	0.5817	0.057*	
C11	0.58717 (16)	0.75136 (6)	0.54189 (17)	0.0387 (3)	
C12	0.73314 (17)	0.70263 (6)	0.59332 (19)	0.0441 (3)	

### Atomic displacement parameters (Å^2^)

**Table:**

	*U*^11^	*U*^22^	*U*^33^	*U*^12^	*U*^13^	*U*^23^
O1	0.0538 (6)	0.0424 (6)	0.0806 (8)	−0.0112 (4)	0.0217 (5)	−0.0014 (5)
O2	0.0410 (6)	0.0627 (7)	0.1006 (9)	−0.0031 (5)	0.0027 (6)	−0.0102 (6)
N1	0.0894 (10)	0.0504 (7)	0.0696 (9)	−0.0025 (7)	0.0429 (8)	−0.0105 (6)
N2	0.0453 (6)	0.0332 (5)	0.0525 (6)	0.0058 (4)	0.0200 (5)	0.0031 (5)
N3	0.0417 (6)	0.0354 (6)	0.0464 (6)	0.0046 (4)	0.0141 (5)	−0.0016 (4)
C1	0.0633 (9)	0.0393 (7)	0.0639 (9)	−0.0074 (6)	0.0273 (7)	−0.0080 (6)
C2	0.0676 (10)	0.0490 (8)	0.0787 (11)	0.0133 (7)	0.0439 (9)	0.0093 (8)
C3	0.0412 (8)	0.0447 (7)	0.0723 (10)	0.0066 (6)	0.0200 (7)	0.0079 (7)
C4	0.0543 (8)	0.0416 (7)	0.0480 (7)	0.0114 (6)	0.0184 (6)	0.0063 (6)
C5	0.0440 (7)	0.0390 (7)	0.0411 (7)	−0.0014 (5)	0.0173 (5)	0.0002 (5)
C6	0.0425 (7)	0.0377 (6)	0.0353 (6)	0.0014 (5)	0.0122 (5)	−0.0005 (5)
C7	0.0437 (8)	0.0503 (8)	0.0565 (8)	0.0053 (6)	0.0090 (6)	−0.0003 (6)
C8	0.0590 (9)	0.0540 (9)	0.0558 (9)	0.0200 (7)	0.0054 (7)	0.0026 (7)
C9	0.0767 (11)	0.0362 (7)	0.0453 (7)	0.0095 (7)	0.0095 (7)	0.0013 (6)
C10	0.0610 (9)	0.0378 (7)	0.0421 (7)	−0.0048 (6)	0.0109 (6)	−0.0034 (5)
C11	0.0448 (7)	0.0378 (7)	0.0339 (6)	0.0002 (5)	0.0111 (5)	−0.0029 (5)
C12	0.0428 (7)	0.0439 (7)	0.0444 (7)	−0.0018 (6)	0.0095 (6)	−0.0055 (5)

### Geometric parameters (Å, °)

**Table:**

O1—C5	1.2030 (15)	C4—H4A	0.9700
O2—C12	1.2026 (15)	C4—H4B	0.9700
N1—C1	1.3075 (18)	C5—C6	1.4861 (17)
N1—C2	1.373 (2)	C6—C7	1.3764 (18)
N2—C1	1.3455 (17)	C6—C11	1.3843 (17)
N2—C3	1.3683 (16)	C7—C8	1.386 (2)
N2—C4	1.4487 (16)	C7—H7A	0.9300
N3—C5	1.3957 (17)	C8—C9	1.377 (2)
N3—C12	1.3996 (16)	C8—H8A	0.9300
N3—C4	1.4496 (15)	C9—C10	1.3845 (19)
C1—H1B	0.9300	C9—H9A	0.9300
C2—C3	1.341 (2)	C10—C11	1.3810 (18)
C2—H2A	0.9300	C10—H10A	0.9300
C3—H3A	0.9300	C11—C12	1.4820 (18)
			
Cg1···Cg1^i^	3.8831 (9)	Cg3···Cg2^iii^	3.6985 (8)
Cg2···Cg3^ii^	3.6985 (8)	Cg3···Cg3^ii^	3.7214 (8)
Cg2···Cg3^iii^	3.6469 (8)	Cg3···Cg3^iii^	3.7214 (8)
Cg3···Cg2^ii^	3.6469 (8)		
			
C1—N1—C2	104.28 (13)	O1—C5—C6	130.18 (13)
C1—N2—C3	106.40 (12)	N3—C5—C6	105.53 (10)
C1—N2—C4	126.73 (12)	C7—C6—C11	121.21 (12)
C3—N2—C4	126.86 (12)	C7—C6—C5	130.44 (12)
C5—N3—C12	112.14 (10)	C11—C6—C5	108.34 (11)
C5—N3—C4	123.97 (11)	C6—C7—C8	117.36 (13)
C12—N3—C4	123.78 (11)	C6—C7—H7A	121.3
N1—C1—N2	112.51 (13)	C8—C7—H7A	121.3
N1—C1—H1B	123.7	C9—C8—C7	121.47 (13)
N2—C1—H1B	123.7	C9—C8—H8A	119.3
C3—C2—N1	110.84 (13)	C7—C8—H8A	119.3
C3—C2—H2A	124.6	C8—C9—C10	121.27 (13)
N1—C2—H2A	124.6	C8—C9—H9A	119.4
C2—C3—N2	105.96 (13)	C10—C9—H9A	119.4
C2—C3—H3A	127.0	C11—C10—C9	117.21 (13)
N2—C3—H3A	127.0	C11—C10—H10A	121.4
N2—C4—N3	111.91 (10)	C9—C10—H10A	121.4
N2—C4—H4A	109.2	C10—C11—C6	121.47 (12)
N3—C4—H4A	109.2	C10—C11—C12	130.15 (12)
N2—C4—H4B	109.2	C6—C11—C12	108.38 (11)
N3—C4—H4B	109.2	O2—C12—N3	124.54 (12)
H4A—C4—H4B	107.9	O2—C12—C11	129.87 (13)
O1—C5—N3	124.30 (12)	N3—C12—C11	105.59 (11)

Symmetry codes: (i) −*x*+2, −*y*+1, −*z*+1; (ii) *x*, −*y*+1/2, *z*−3/2; (iii) *x*, −*y*+1/2, *z*−1/2.

### Hydrogen-bond geometry (Å, °)

**Table:**

*D*—H···*A*	*D*—H	H···*A*	*D*···*A*	*D*—H···*A*
C10—H10A···Cg1^iv^	0.93	2.94	3.6105 (15)	130

Symmetry codes: (iv) −*x*+1, *y*−1/2, −*z*+1/2.
